# Antioxidant, α-glucosidase inhibitory activity and sub-chronic toxicity of *Derris reticulata* extract: its antidiabetic potential

**DOI:** 10.1186/s12906-015-0552-4

**Published:** 2015-02-27

**Authors:** Pakarang Kumkrai, Oratai Weeranantanapan, Nuannoi Chudapongse

**Affiliations:** School of Pharmacology, Institute of Science, Suranaree University of Technology, Nakhon Ratchasima, 30000 Thailand; School of Anatomy, Institute of Science, Suranaree University of Technology, Nakhon Ratchasima, 30000 Thailand; Current address: Division of Health Promotion, Faculty of Health Science, Srinakharinwirot University, Ongkharak, Nakhon-Nayok 26120 Thailand

**Keywords:** *Derris reticulata*, Antidiabetes, Antioxidant, α-Glucosidase, RINm5F cells

## Abstract

**Background:**

Antidiabetic activity of *Derris reticulata* extract on alloxan-induced diabetic rats has been reported. The extract was found to lower blood glucose and inhibit intestinal glucose absorption. The aim of this study was to further investigate mechanisms underlying the antihyperglycemic activity of *D. reticulata* extract *in vitro*.

**Methods:**

The aqueous extract was obtained from *D. reticulata* stem. Phytochemical screening, total phenolic, and flavanoid contents were examined. ABTS and DPPH scavenging assays, and FRAP method were used to determine *in vitro* antioxidant activities. Measurement of cell viability on alloxan-induced cellular damage was performed in the insulin-secreting RINm5F cells by MTT assay. The effects of the extract on α-glucosidase activity and insulin release were studied. In addition, sub-chronic toxicity test in rats was also conducted.

**Results:**

The results revealed that the extract, which consisted of terpenoids, saponins, tannins and flavonoids, possessed moderate radical scavenging activities. Pre-treatment of RINm5F cells with the extract was also found to exert moderate, but significant, *in vitro* protection against alloxan, an oxidative stress producing agent. Unlike glibenclamide, the extract did not stimulate insulin secretion. However, the extract was found to inhibit α-glucosidase activity similar to acarbose. It was found that in sub-chronic toxicity studies *D. reticulata* extract did not cause mortality or produce any remarkable haematological, biochemical and histopathological adverse effects in rats.

**Conclusions:**

The data suggest that the possible mechanisms underlying antihyperglycemic activity of *D. reticulata* extract are cytoprotective effect on pancreatic cells, presumably by its antioxidant activity, and inhibition of α-glucosidase. Sub-chronic toxicity study also provides scientific evidence to corroborate the safety of this plant as an alternative antidiabetic agent.

## Background

Diabetes mellitus is a chronic metabolic disease characterized by hyperglycemia resulting from reduction of insulin secretion and/or insulin resistance. It is widely known that elevation of blood glucose caused by disruption of carbohydrate, protein and fat metabolism can lead to diabetic complications in several organs and tissues, including eyes, kidneys, nerves and blood vessels [1]. It has been shown that current therapy for diabetes with synthetic hypoglycemic agents can produce adverse effects such as hypoglycemia, gastrointestinal disturbances and hepatotoxicity [2]. Therefore, the search for herbal formulation with antidiabetic activity remains a topic of research interest.

Plants from diverse families have been shown to possess potent hypoglycemic activities [3]. Thus, several plants have been used as an alternative treatment in patients with diabetes [4]. In Thailand, the use of traditional medicine plays an important role in public health care and many medicinal plants have been used to treat several ailments including diabetes [5]. In our previous report, the efficacy of the aqueous extract of *Derris reticulata* stems, a local plant used by some rural Thais to treat diabetes, has been demonstrated in alloxan-induced diabetic rats [6]. In this report, we further investigated antioxidant activity of *D. reticulata* extract to support its cytoprotective potential. The effects of the extract on α-glucosidase activity and insulin release were studied. In addition, sub-chronic toxicity test was also conducted to establish the safety of the extract in rats.

## Methods

### Collection of plant material and extraction

Plant collection and extraction were performed as previously described by our group [6]. Briefly, small pieces of dried stem (100 g) were boiled twice with 500 ml of distilled water for 10 min each. After filtered through cotton gauze, the filtrate was centrifuged at 2,500 × g for 10 min. Supernatant was collected and lyophilized. The dried extract (yield 16.73%, w/w) was kept at −20°C until used. Authenticity of the plant was verified by Dr. Paul J. Grote and the voucher specimen (Pharm-Chu-006) was deposited at School of Pharmacology, Suranaree University of Technology (SUT).

### Phytochemical screening

Phytochemical screening was carried out to identify constituents of *D. reticulata* extract. The extract was screened for anthraquinones, terpenoids, flavonoids, saponins, tannins and cardiac glycosides. Phytochemical screening was performed based on previously reported methods [7,8].

### Determination of antioxidant compounds

#### Determination of total phenolic content

The phenolic compounds of the *D. reticulata* extract was determined by a method previously described [9]. *D. reticulata* extract (5 mg) was dissolved in 1 ml of distilled water. A 100 μl of aliquot was mixed with 2 ml of 2% Na_2_CO_3_. The mixture was left standing for 2 min at room temperature followed by an addition of 100 μl of Folin-Ciocalteau reagent (diluted with methanol 1:1 v/v). After incubation for 30 min, absorbance was measured at 750 nm using spectrophotometer. Gallic acid was used for standard curve calibration. Total phenolic content of *D. reticulata* extract was expressed as mg gallic acid equivalents (GAE) per gram extract.

#### Determination of total flavonoid content

Total flavonoid content was measured according to the method reported by Liu and coworkers [10]. In brief, 250 μl of *D. reticulata* extract (5 mg/ml) was diluted with 1250 μl of distilled water. 75 μl of a 5% NaNO_2_ solution was added to the mixture and incubated for 6 min. After the incubation period, 150 μl of 10% AlCl_3_ solution was added. The mixture was further incubated for 5 min. Then, 500 μl of 1 M NaOH was added, and the final volume was adjusted to 2500 μl with distilled water. The absorbance was measured at 510 nm and compared to standard catechin. Total flavonoid content of *D. reticulata* extract was expressed as mg catechin per gram extract.

### Determination of antioxidant activities

#### ABTS (2,2′-azino-bis(3-ethylbenzothiazoline-6-sulfonate) scavenging assay

The radical scavenging activity of *D. reticulata* extract against ABTS^•+^ was carried out according to the procedure described previously [11]. Briefly, ABTS^•+^ radical cation was produced by mixing 5 ml of 14 mM ABTS with 5 ml of 4.9 mM potassium persulphate (K_2_S_2_O_8_) for 16 h in the dark at room temperature. Before used, the ABTS^•+^ solution was diluted with ethanol to an absorbance of 0.70 ± 0.02 at 734 nm. A various concentrations (50 μl) of *D. reticulata* extract was mixed with 950 μl of diluted ABTS^•+^ solution. After 6 min of incubation, the absorbance was read at 734 nm. Butylated hydroxytoluene (BHT) was used as standard. The antioxidant activity was expressed as IC_50_ (the concentration required for 50% scavenging of free radical).

#### DPPH (2,2-diphenyl-1-picrylhydrazyl) scavenging assay

The ability of the *D. reticulata* extract to scavenge DPPH free radical was measured according to the procedure described previously [12] with slight modifications. A various concentrations (1 ml) of the *D. reticulata* extract were added to 4 ml of DPPH methanolic solution (final concentration of DPPH: 0.2 M). The reaction mixture was shaken and left standing at room temperature for 30 min in the dark and then spectroscopically measured at 517 nm. The antioxidant activity was expressed as IC_50_ similar to the ABTS scavenging method.

#### Ferric reducing antioxidant power (FRAP) assay

The ferric reducing power of the extract was performed according to the procedure described previously [13]. FRAP reagent consists of 10 mM TPTZ solution (2,4,6-tripyridyl-s-triazine) in 40 mM HCl, 20 mM FeCl_3_, and 300 mM acetate buffer (pH 3.6), in ratio 1:1:10 (v/v/v), respectively. FRAP reagent was freshly prepared and incubated at 37°C until used. The reaction mixtures were composed of 50 μl of *D. reticulata* extract (5 mg/ml) mixed with 1.5 ml of the FRAP reagent. After 4 min, the absorbance was measured at 593 nm. The reducing potential of the *D. reticulata* extract was determined from a standard curve of FeSO_4_ and the FRAP value was expressed as μmol Fe^2+^/mg dried extract.

### Determination of cell viability on alloxan-induced cellular damage

RINm5F, *Rattus norvegicus* (rat) cell line was obtained from the American Type Culture Collection (ATCC, Manassas, USA). RINm5F cells were cultured in RPMI-1640 medium supplemented with 10% fetal bovine serum, 1% antibiotic-antimycotic solution and incubated at 37°C in a humidified atmosphere containing 5% CO_2_.

RINm5F cells (2 × 10^5^) were incubated with *D. reticulata* extract for 23 h. After the incubation period, the medium was removed. Cells were treated with alloxan at 9 mM (which caused about 50% of cell death) for 1 h. At the end of experiment, cell viability was assessed by MTT assay as previously described [14].

### Determination of α-glucosidase inhibitory effect

The α-glucosidase inhibitory activity was measured as described previously [15] with minor changes. The crude enzyme solution was prepared from *Saccharomyces cerevisiae Type* I (Sigma–Aldrich, USA). The reaction mixture consisted of crude enzyme solution (0.1 U/ml, 10 μl), in 0.1 M potassium phosphate buffer pH 6.8 (50 μl), and the test sample (20 μl). Acarbose (Bayer, Indonesia) was used as positive control. After pre-incubation at 37°C for 10 min, 10 μl of 1 mM *p*-nitrophenyl-α-D-glucopyranoside (Sigma–Aldrich, USA) was added. The solution was incubated for an additional 30 min at 37°C. The reaction was terminated by adding 50 μl of 0.1 M Na_2_CO_3_. At the end of incubation, the absorbance was measured at 405 nm. The concentration of inhibitors required for inhibiting 50% of the α-glucosidase activity under the assay conditions was defined as the IC_50_ value.

### Determination of the effect on insulin secretion

The insulin secretion assay was performed according to the previously described method [16]. RINm5F cells (2 × 10^5^ cells/well) were seeded in 96 well plates and grown to reach 70-80% confluent state. Culture medium was removed and replaced with Kreb’s Ringer buffer. After 60 min of incubation, cells were washed twice with fresh Kreb’s Ringer buffer and then incubated with the *D. reticulata* extract at 250 and 500 μg/ml, and glibenclamide (50 μg/ml) for 60 min. The extract was diluted with Kreb’s Ringer buffer. Glibenclamide was dissolved in DMSO and further diluted with Kreb’s Ringer buffer. The final concentration of DMSO (0.5% w/v) did not affect RINm5F cell viability. The supernatant was collected for measurement of insulin release by enzyme-linked immunosorbent assay (ELISA).

### Sub-chronic toxicity study

Male and female Wistar rats used in this study were obtained from Laboratory Animal Center, SUT. Animals were acclimatized for 7 days prior to the experiments. The rats were housed in polypropylene cages, with free access to normal diet and water *ad libitum*. The rats were maintained at room temperature (25 ± 0.5°C), relative humidity 45-50% and at 12 h light/dark cycle. All procedures in this study were approved and conducted according to guidelines of the Institutional Animal Care and Use Committee, SUT. All efforts were made to minimize the number of rats used and their suffering.

Wistar rats of both sexes were randomly assigned into five groups, a control and four treatment groups (*n* = 8; 4 males and 4 females). The treatment groups were given the extract 250, 500, 1000 and 2000 mg/kg by oral route once a day for 42 days. The body weights were recorded at the end of each week. All animals were observed daily for clinical signs and mortality throughout the treatment period. At the end of treatment, rats were fasted overnight for 14 h. All rats were anesthetized by CO_2_ inhalation and blood samples were immediately collected via cardiac puncture for haematological and biochemical analyses. After blood collection, the rats were sacrificed by cervical dislocation. Internal organs were excised, weighed, and examined macroscopically. The relative organ weight was calculated as (organ weight/body weight) × 100%. Vital organs such as liver and kidney were preserved for histopathological examinations.

#### Haematological and biochemical evaluations

Haematological analysis was determined using a blood autoanalyzer Coulter (Beckman Coulter Inc., Ireland). The measured haematological parameters included: red blood cell (RBC), white blood cell (WBC), lymphocyte (LYM), monophil (MON), eosinophil (EOS), basophil (BAS), platelet cell (PLT), haemoglobin (HGB), haematocrit (HCT), mean corpuscular volume (MCV), mean corpuscular haemoglobin (MCH) and mean corpuscular haemoglobin concentration (MCHC).

For biochemical parameters analysis, blood sample without anticoagulant was centrifuged at 3,000 × g for 5 min to obtain serum. Serum was stored at −20°C until analysis. The serum was analysed by A15 Analyzer Automatic Clinical Chemistry (Biosystems S.A., Spain). Biochemical parameters assessed included: glucose, total cholesterol, triglyceride, creatinine, aspartate transaminase (AST), alanine transaminase (ALT).

#### Histopathological examinations

Tissue samples of liver and kidney were fixed with 10% neutral buffered formaldehyde and dehydrated in serial ethanol solution (70%, 95% and 100%) and acetone. Tissue samples were cleaned with xylene. The tissues were infiltrated with molten paraffin at 65°C and embedded in paraffin block and sectioned at 5 μm thickness. The sections were mounted onto slides using gelatin coating solution and dried at 56°C for 45 min in a hot air oven. Tissue sections were deparaffined in xylene and were hydrated with serial ethanol solution (100%, 95% and 70%). The sections were washed in running tap water, and stained with haematoxylin for 10 min and washed in running tap water. The sections were stained with eosin for 10 min, followed by dehydrated with serial ethanol solution (70%, 95% and 100%). Finally, the sections were immersed in acetone and xylene and covered with cover slip after drying. The photomicrographs of each tissue section were observed under microscope (Olympus, Japan).

### Statistical analysis

Data are expressed as mean ± S.E.M. Comparisons among different groups were performed by analysis of variance (ANOVA) followed by Student-Newman-Keuls test. *P*-values less than 0.05 were set as the level of significance.

## Results

### Phytochemical compositions, phenolic content and total flavonoid content

The extract of *D. reticulata* stem obtained from this study contained terpenoids, flavonoids, saponins and tannins but not anthraquinones and cardiac glycosides. Total phenolic content and total flavonoid content found in *D. reticulata* extract were 78.84 ± 0.01 mg GAE/g extract and 54.72 ± 1.81 mg catechin/g extract, respectively.

### Antioxidant activity of *D. reticulata* extract

It has been demonstrated that *in vitro* analytical methods are reliable determination of antioxidant activity of biological sample. However, it has been recommended that assays based on hydrogen atom transfer and electron transfer reaction together should be used to provide a better view of antioxidant activity than a single method [17]. Accordingly, in this study, three analysis methods including ABTS radical scavenging, DPPH radical scavenging and ferric reducing antioxidant power (FRAP) assays were performed to determine and confirm antioxidant activity of *D. reticulata* extract. As shown in Table 1, the IC_50_ of ABTS radical scavenging activity of the extract was found at 515.05 ± 0.13 μg/ml, whereas that of DPPH scavenging activity was at 239.85 ± 0.13 μg/ml. In addition, the FRAP value of the extract was 0.23 ± 0.05 μmol Fe^2+^/mg dried extract.

**Table 1:** **Antioxidant activities of** ***Derris reticulata*** **extract**

	**Methods**		
	**ABTS**	**DPPH**	**FRAP**
	**(IC**_**50**_ **: μg/ml)**	**(IC**_**50**_ **: μg/ml)**	**(μmol Fe** ^**2+**^ **/mg dried extract)**
*Derris reticulata* extract	515.05 ± 0.13	239.85 ± 0.13	0.23 ± 0.05
Ascorbic acid	-	1.23 ± 0.18	-
Butylated hydroxytoluene (BHT)	83.05 ± 0.13	-	-

Values are expressed as means ± S.E.M. (n = 3).

### *In vitro* cytoprotective effect of the extract

**Figure 1:**
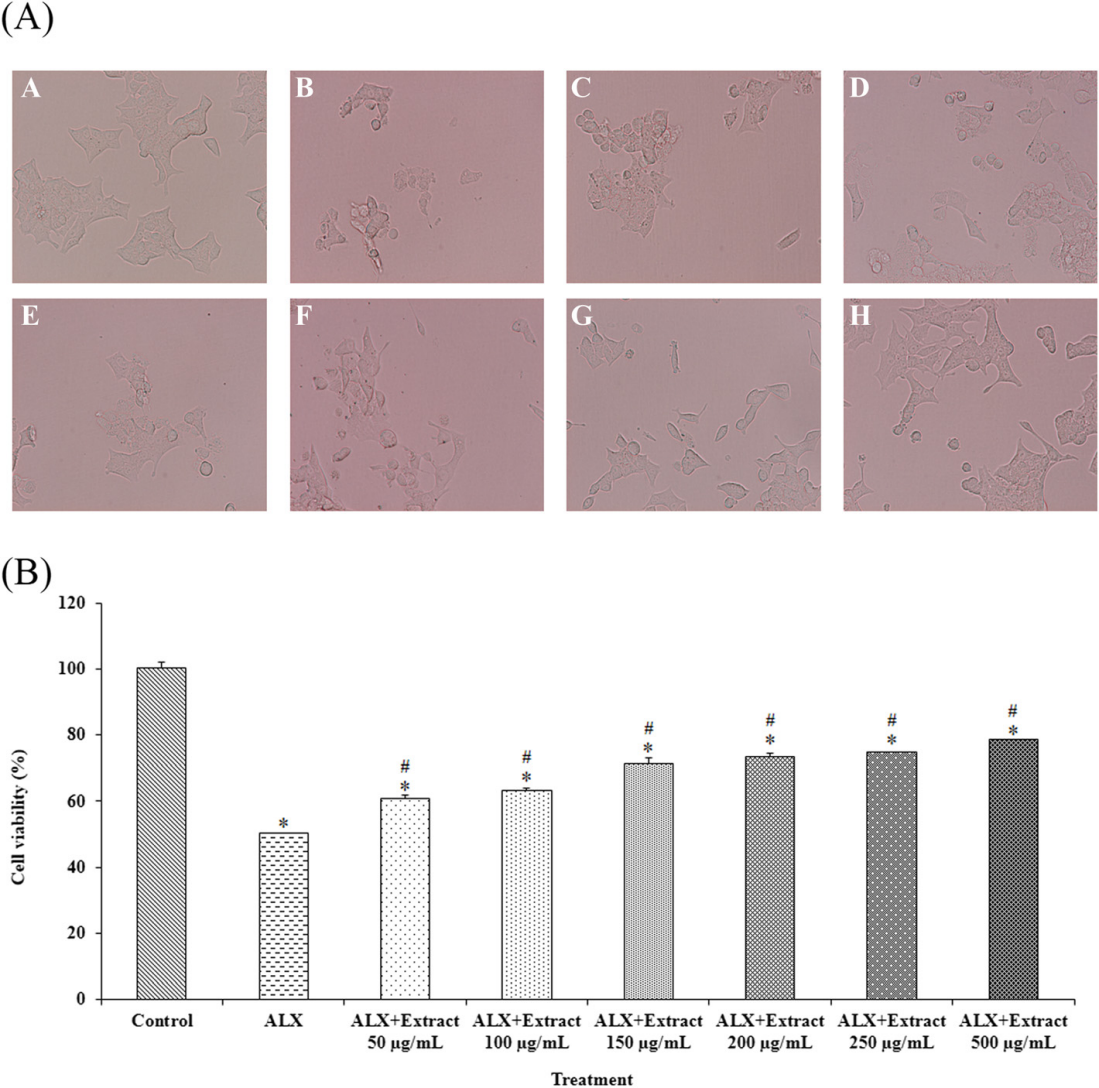
**Cytoprotective effect of** ***D. reticulata*** **extract on cell viability.** Panel **(A)** is microscopic images (200×) of RINm5F cells whereas Panel **(B)** is the effect on % cell viability measured by MTT assay. Pre-treatment with *D. reticulata* extract was performed on alloxan-induced RINm5F cell damage. The IC_50_ of alloxan (9 mM) was used to induce cell death after pre-treatment with various concentrations of the extract. In panel **(A)**, A is normal control whereas B is cells treated with alloxan alone. The cells in panels C-H were treated with the extract at 50, 100, 150, 200, 250 and 500 μg/ml, respectively, for 23 hours before exposure of alloxan. Values in Panel **(B)** are expressed as mean ± S.E.M. (n = 3). * *p* < 0.05 statistically significant difference compared to control. # *p* < 0.05 statistically significant difference compared to alloxan (ALX).

The protective effect of *D. reticulata* extract against alloxan-induced cell death was studied by MTT assay. The half maximal inhibitory concentration (9 mM) of alloxan on cell viability was used for studying the cytoprotective effect of the extract. It was found that alloxan, a free radical producing agent, decreased the number of RINm5F cells and altered their morphology as well as caused cell detachment from plate (Figure 1A). In accordance with microscopic observation, the result from MTT assay (Figure 1B) showed that pretreatments of *D. reticulata* extract provided a significant protection effect from alloxan-induced RINm5F cell damage. The extract increased cell viability from 50.25 ± 0.30 up to 78.51 ± 0.29%. Note that the concentration of the extract was limited at 500 μg/ml to avoid toxicity on cell viability that may be caused by the extract itself.

### Inhibitory effect of ***D. reticulata*** extract on α-glucosidase activity

**Figure 2:**
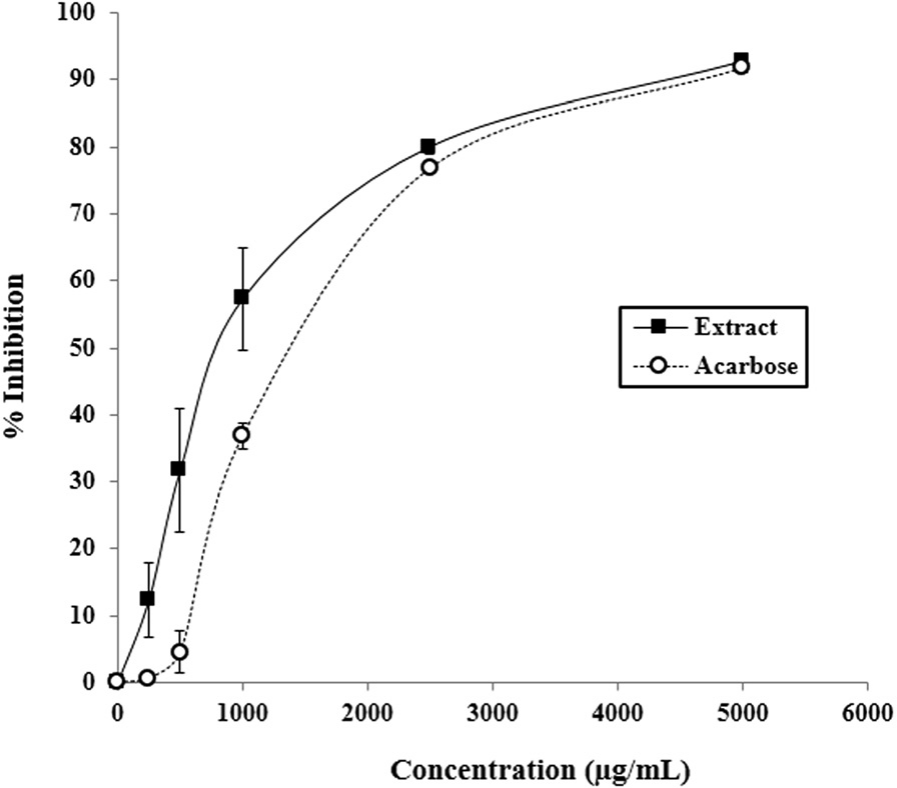
**Inhibitory effect of** ***D. reticulata*** **extract on α-glucosidase activity.** Values are expressed as mean ± S.E.M. of three separate experiments. Acarbose was used as positive reference. The calculated IC_50_ of the extract was 918 ± 172 μg/ml whereas that of acarbose was 1379 ± 17 μg/ml.

Several concentrations (250–5000 μg/ml) of *D. reticulata* extract were tested to determine levels of α-glucosidase inhibition. It was found that *D. reticulata* extract inhibited α-glucosidase activity in dose-dependent manner with the IC_50_ of 917.29 ± 0.13 μg/ml, whereas the IC_50_ of acarbose (positive control) was 1378.99 ± 0.13 μg/ml (Figure 2). The maximal inhibition of the extract reached approximately 92% similar to that of acarbose.

### Effect of *D. reticulata* extract on insulin secretion

**Figure 3:**
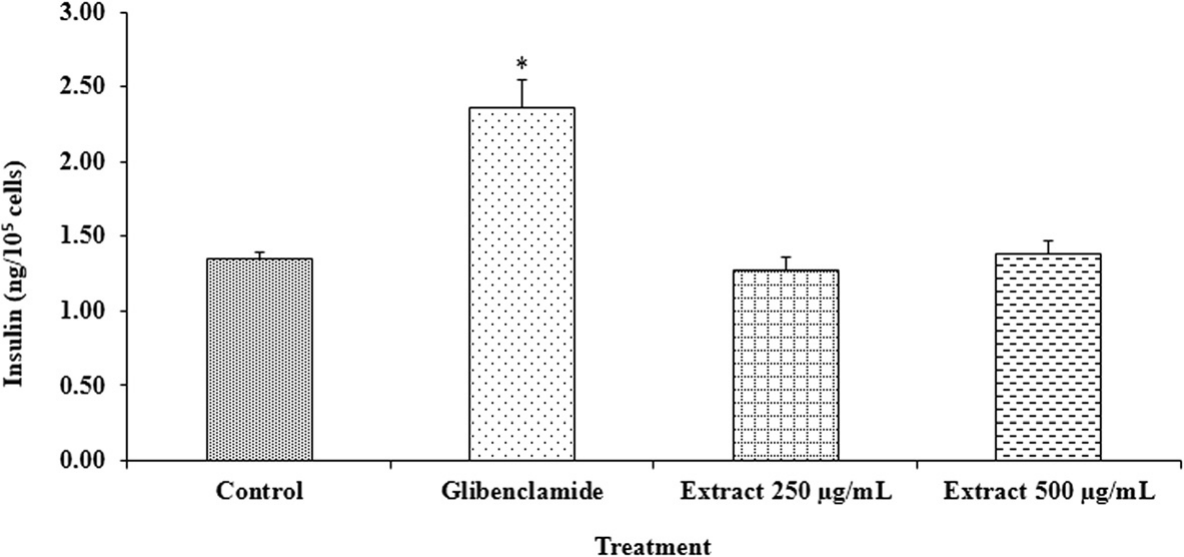
**Effect of** ***D. reticulata*** **extract on insulin secretion.** Glibenclamide significantly increased insulin concentration in the medium from 1.35 ± 0.04 (control) to 2.36 ± 0.15 ng/10^5^ cells. Treatment with the extract at the doses of 250 and 500 μg/ml did not significantly alter insulin secretion from RINm5F cells (1.27 ± 0.09 and 1.38 ± 0.09 ng/10^5^ cells, respectively). Values are expressed as mean ± S.E.M. (n = 3). * *p* < 0.05 statistically significant difference from control.

RINm5Fcells were used to evaluate the effect of *D. reticulata* extract on insulin secretion *in vitro* and the data were shown in Figure 3. The insulin secretagogue glibenclamide significantly increased insulin concentration in the medium from 1.35 ± 0.04 to 2.36 ± 0.15 ng/10^5^ cells compared to control. It was found that unlike glibenclamide, the extract at the doses of 250 and 500 μg/ml did not significantly alter insulin secretion from RINm5F cells.

### Sub-chronic toxicity

#### Clinical observation, body weight and relative organ weight

Sub-chronic administration of *D. reticulata* extract did not produce clinical signs of toxicity. The body weight increased throughout the study period in male and female rats of both control and treated groups. The patterns of water and food consumption were similar in all groups of animals. No lethality was observed during the 42-day period of treatment with *D. reticulata* extract. There were no significant changes in the relative organ weight of all animals treated with *D. reticulata* extract at various doses as compared to the control group.

#### Haematological and biochemical parameters

Haematological parameters of male and female rats depicted in Tables 2 and 3 showed that the number of red blood cells and white blood cells of treatment groups were not significantly different from the control group. However, the number of platelets of all treated female rats significantly increased (Table 2) while male rats treated with *D. reticulata* extract at 250 mg/kg had a significant decrease of MCH (Table 3). However, this effect was not dose dependent because it was not found in the groups treated with 500, 1000 and 2000 mg/kg of *D. reticulata* extract. There were significant differences (*p* < 0.05) in MCV and MCHC between the treated and the control groups in male rats. More importantly, it should be noted that all of the parameters in treated groups that were different compared to control are within the normal ranges. Biochemical parameter profiles of male and female rats are presented in Table 4. Oral administration of *D. reticulata* extract did not cause significant changes in the level of glucose, total cholesterol, triglyceride, creatinine, AST and ALT. All biochemical parameters analyzed were within the normal laboratory ranges. Thus the extract did not cause abnormality in the haematological and biochemical parameters of rats.

**Table 2:** **Haematological parameters of female rats administered with** ***Derris reticulata*** **extract for 42 days**

**Parameters**	**Control**	**Treatment**			
		**250 mg/kg**	**500 mg/kg**	**1000 mg/kg**	**2000 mg/kg**
***Female***					
RBC (×10^6^/μl)	8.10 ± 0.17	7.94 ± 0.32	8.21 ± 0.19	8.12 ± 0.15	8.40 ± 0.16
HGB (g/dl)	16.00 ± 0.00	15.50 ± 0.65	16.25 ± 0.25	16.00 ± 0.00	16.75 ± 0.25
HCT (%)	45.50 ± 0.65	43.25 ± 2.10	44.00 ± 0.71	44.50 ± 0.87	45.00 ± 0.82
WBC (×10^3^/μl)	1.33 ± 0.25	5.18 ± 2.26	1.08 ± 0.40	1.35 ± 0.35	1.58 ± 0.11
LYM (%)	86.25 ± 0.95	87.50 ± 3.40	76.25 ± 4.63	82.25 ± 4.84	88.50 ± 1.04
MON (%)	2.00 ± 0.41	3.50 ± 1.56	2.00 ± 0.71	1.75 ± 1.11	0.75 ± 0.48
EOS (%)	2.25 ± 0.85	1.00 ± 1.00	5.50 ± 4.27	3.50 ± 2.22	1.50 ± 0.29
BAS (%)	0.00 ± 0.00	0.00 ± 0.00	0.00 ± 0.00	0.00 ± 0.00	0.00 ± 0.00
PLT (10^3^/μl)	618.5 ± 43.1	811.8 ± 34.8*	807.3 ± 49.0*	818.8 ± 43.3*	752.8 ± 45.2*
MCV (fl)	56.00 ± 0.58	54.43 ± 0.80	53.78 ± 0.60	54.80 ± 0.46	53.63 ± 0.29
MCH (pg)	19.75 ± 0.40	19.68 ± 0.20	19.63 ± 0.34	19.78 ± 0.14	19.60 ± 0.06
MCHC (g/dl)	35.28 ± 0.55	36.10 ± 0.27	36.50 ± 0.27	36.08 ± 0.27	36.55 ± 0.25

Values are expressed as mean ± S.E.M. (n = 4/group).

*p < 0.05 statistically significant difference from control.

Note that all of the parameters in treated groups that were different compared to control are within the normal ranges.

**Table 3:** **Haematological parameters of male rats administered with** ***Derris reticulata*** **extract for 42 days**

**Parameters**	**Control**	**Treatment**			
		**250 mg/kg**	**500 mg/kg**	**1000 mg/kg**	**2000 mg/kg**
***Male***					
RBC (×10^6^/μl)	9.15 ± 0.21	9.55 ± 0.11	8.56 ± 0.24	8.82 ± 0.28	8.80 ± 0.16
HGB (g/dl)	17.75 ± 0.25	17.50 ± 0.29	16.50 ± 0.50	17.25 ± 0.63	16.75 ± 0.25
HCT (%)	52.50 ± 1.56	50.25 ± 0.63	47.00 ± 1.68	48.00 ± 2.12	47.00 ± 0.91
WBC (×10^3^/μl)	1.48 ± 0.28	5.58 ± 1.84	4.50 ± 1.43	3.80 ± 0.38	2.15 ± 0.70
LYM (%)	83.50 ± 2.96	75.50 ± 5.72	85.00 ± 2.48	85.50 ± 1.66	82.50 ± 5.12
MON (%)	3.25 ± 2.02	3.25 ± 1.11	2.75 ± 1.44	2.75 ± 0.85	2.00 ± 1.00
EOS (%)	0.25 ± 0.25	0.50 ± 0.29	0.75 ± 0.25	0.50 ± 0.29	0.75 ± 0.75
BAS (%)	0.00 ± 0.00	0.00 ± 0.00	0.00 ± 0.00	0.00 ± 0.00	0.00 ± 0.00
PLT (10^3^/μl)	731.3 ± 45.3	863.0 ± 44.5	638.8 ± 35.7	667.8 ± 21.4	798.8 ± 43.5
MCV (fl)	57.10 ± 0.58	52.63 ± 0.37*	54.90 ± 0.38*	54.30 ± 0.86*	53.45 ± 0.43*
MCH (pg)	19.25 ± 0.24	18.35 ± 0.10*	19.40 ± 0.14	19.18 ± 0.18	19.03 ± 0.11
MCHC (g/dl)	33.65 ± 0.30	34.88 ± 0.17*	35.38 ± 0.29*	35.28 ± 0.26*	35.63 ± 0.36*

Values are expressed as mean ± S.E.M. (n = 4/group).

*p < 0.05 statistically significant difference from control.

Note that all of the parameters in treated groups that were different compared to control are within the normal ranges.

**Table 4:** **Biochemical parameters of male and female rats administered with** ***Derris reticulata*** **extract for 42 days**

**Parameters**	**Control**	**Treatment**			
		**250 mg/kg**	**500 mg/kg**	**1000 mg/kg**	**2000 mg/kg**
***Male***					
Glucose (mg/dl)	125.8 ± 10.4	114.3 ± 10.7	135.8 ± 5.1	114.0 ± 2.0	118.5 ± 4.3
Total cholesterol (mg/dl)	61.50 ± 2.60	52.75 ± 3.50	61.50 ± 4.52	44.50 ± 2.84	50.75 ± 5.82
Triglyceride (mg/dl)	80.75 ± 18.44	85.50 ± 6.61	81.75 ± 13.83	76.75 ± 6.16	93.75 ± 23.02
Creatinine (mg/dl)	0.78 ± 0.09	0.65 ± 0.03	0.69 ± 0.04	0.68 ± 0.01	0.67 ± 0.04
AST (U/l)	96.25 ± 7.39	96.00 ± 6.49	99.00 ± 9.46	95.00 ± 10.98	98.00 ± 8.37
ALT (U/l)	32.75 ± 4.27	28.25 ± 3.64	33.25 ± 2.93	25.50 ± 2.72	29.25 ± 3.09
***Female***					
Glucose (mg/dl)	101.0 ± 8.2	91.00 ± 2.1	104.5 ± 7.0	101.8 ± 7.0	83.75 ± 4.4
Total cholesterol (mg/dl)	53.00 ± 2.80	45.50 ± 3.20	45.75 ± 4.64	44.25 ± 1.49	42.25 ± 5.47
Triglyceride (mg/dl)	40.50 ± 10.21	47.25 ± 3.50	52.25 ± 14.63	35.75 ± 2.87	47.75 ± 7.12
Creatinine (mg/dl)	0.74 ± 0.06	0.83 ± 0.06	0.73 ± 0.04	0.74 ± 0.07	0.76 ± 0.02
AST (U/l)	93.75 ± 6.21	86.00 ± 5.87	88.00 ± 7.36	75.00 ± 5.45	86.00 ± 4.06
ALT (U/l)	24.25 ± 2.50	35.25 ± 13.33	23.75 ± 1.55	19.25 ± 0.48	23.00 ± 1.47

Values are expressed as mean ± S.E.M. (*n* = 4/group).

There was no significant difference among control and treatment groups (*p* > 0.05).

#### Histopathological examinations

**Figure 4:**
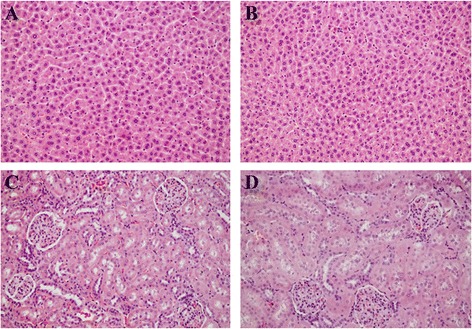
**Representative photomicrographs of liver and kidney of male and female rats after administered with the aqueous extract of** ***D. reticulata.*** **A**: Liver control; **B**: Liver of animal treated with 2000 mg/kg extract; **C**: Kidney control; **D**: Kidney of animal treated with 2000 mg/kg extract. Hematoxylin and eosin staining (200×).

There was no macroscopic change of internal organs (i.e., appearance, color, size) considered to be related to the treatment. The histopathological examinations of liver and kidney revealed no morphological alteration in all treatment and control groups. Representative photomicrographs of liver and kidney were shown in Figure 4.

## Discussion

Phenolic compounds are secondary metabolites of plants, which widely distributed throughout the plant kingdom. Phenolics are gaining attention because their antioxidant activities have shown health benefits [18,19]. Plant phenolics have exhibited health protective effects in many ailments, for example inflammation, cancer and hypertension [20]. The antioxidant activity of plant materials is well correlated with their content of phenolic compounds [21,22].

In this study, the phytochemical qualitative screening tests revealed that *D. reticulata* extract consisted of terpenoids, flavonoids, saponins and tannins but not anthraquinones and cardiac glycosides. Lupinifolin which is a flavonoid compound has been found to be a major compound of *D. reticulata* Craib. [23]. Phytochemical studies of other plants in *Derris* genus have been reported. For example, two pyranoflavanones, epoxylupinifolin and dereticulatin isolated from the stem of *D. reticulata* Benth. were identified [24]. Furanoflavanoids were also isolated from *D. indica* [25] whereas the study of *D. laxiflora* has revealed that it contains some triterpenoids [26]. Scandenin A, scandenin B and isoflavone derivatives have been found in *D. scandens* [27,28].

Total phenolic content determined by Folin-Ciocalteu method was 78.84 ± 0.01 mg GAE/g extract. Antioxidant compounds of *D. reticulata* extract presumably were tannins and flavonoids found in preliminary phytochemical analysis. Total flavonoid content in the extract, as measured by aluminium chloride colorimetric method was 54.72 ± 1.81 mg catechin/g extract. Several studies have reported that flavonoids possess antioxidant property [29,30]; and the hydroxyl groups in flavonoids are responsible for the free radical scavenging activity of these compounds [31].

ABTS radical scavenging, DPPH radical scavenging and ferric reducing antioxidant power (FRAP) assays were performed to determine the antioxidant activity of *D. reticulata* extract. As shown in Table 1, the IC_50_ of ABTS radical scavenging activity of the extract was 515.05 ± 0.13 μg/ml, whereas that of DPPH scavenging activity was 239.85 ± 0.13 μg/ml. In addition, the FRAP value of the extract was 0.23 ± 0.05 μmol Fe^2+^/mg dried extract. Together, the results indicate that *D. reticulata* extract possessed a moderate degree of radical scavenging activities.

The aqueous extract of *D. reticulata* stem was previously reported to possess potential antidiabetic property in rats [6]. The phytochemical analysis of the aqueous extract of *D. reticulata* stem revealed several phenolic constituents which could have potential antidiabetic property as shown in some other herbs. For example, it has been reported that *Solanum torvum* Swartz extract containing phenolic compounds (rutin, caffeic acid, gallic acid and catechin) exhibits hypoglycemic activity and is known for their ability to promote β-cell regeneration [32]. Flavonoids and triterpenoids, the two major types of the compounds found in *Potentilla discolor* extract have protective effects on β-cells in diabetic rats [30]. As shown in Figure 1, pre-treatments with *D. reticulata* extract at the doses of 50–500 μg/ml provided a significant protective effect from alloxan-induced RINm5F cell death. It is known that alloxan induces diabetes mellitus by generating free radical, resulting in its toxic action on pancreatic β-cells [33,34]. In accordance with its moderate antioxidant activity found in this study, pretreatment of *D. reticulata* extract increased cell viability up to only about 78%. The results suggests that the cytoprotective effect of *D. reticulata* extract against alloxan may be partly derived from the free radical scavenging activity of its antioxidant compounds.

Uncontrolled hyperglycemia in diabetic patients is associated with profound complications, such as increased risk of coronary heart disease, peripheral vascular disease, and cerebrovascular disease [1]. The reduction of postprandial hyperglycemia has been approached by suppression of carbohydrate absorption from gastrointestinal tract [35]. Several plant extracts have been shown to exert antidiabetic property through α-glucosidase inhibition, for example, the aqueous extract of *Ficus deltoidea* [22], the acetone leaf extracts of *F. lutea* [36] and the hydro-alcoholic extracts of *Polygonum senegalensis* and *P. kotschyi* [37]. In addition to the interference of intestinal glucose absorption as previously reported [6], it was found in the present study that *D. reticulata* extract also inhibited α-glucosidase *in vitro* with the IC_50_ of 918 ± 172 μg/ml, whereas the IC_50_ of acarbose, an antidiabetic drug known to inhibit intestinal α-glucosidase, was 1379 ± 17 μg/ml (Figure 2). This action should contribute to the anti-hyperglycemic effect of the extract. The result also suggests that the extract may be clinically useful for the control of postprandial hyperglycemia.

RINm5F cells have been used for studying the effect of insulin secretagogues. In this study, the insulin concentrations of the cell culture supernatant were determined. Glibenclamide, an insulin secretagogue, was selected as positive control. Glibenclamide stimulates insulin secretion by blocking ATP-sensitive potassium channels of the β-cell membrane, thereby causing depolarization calcium influx, and rising in cytoplasmic calcium concentration [38]. RINm5F cells treated with glibenclamide showed a significant increase in insulin secretion (Figure 3). In contrast, *D. reticulata* extract at the doses of 250 and 500 μg/ml did not possess a stimulatory effect on insulin release. This result suggests that the extract may have some advantages over glibenclamide or other insulin secretagogues in terms of causing fewer clinical events of hypoglycemia.

Because diabetes mellitus is a chronic metabolic disease which requires long term treatment, its safety data for long term use is crucial. Therefore, sub-chronic toxicity of *D. reticulata* extract was evaluated in rats for 42 days. The extract at the doses of 250, 500, 1000 and 2000 mg/kg did not affect body weight of the treated animals compared to control. Animal behaviors were also observed and the extract did not produce any signs of behavioral toxicity. Organ weight changes have long been accepted as a sensitive indicator of chemically induced organ alterations. It was found that the extract did not induce any changes in relative organ weight. Several haematological and biochemical parameters were analyzed. There were no significant changes of all tested parameters to the toxic level (Tables 2, 3, 4). Histopathological observation on liver and kidney of both control and treatment groups revealed that sub-chronic oral administration of the extract did not cause any alteration of organ morphology (Figure 4). The results indicate that the aqueous extract of *D. reticulata* do not cause mortality or produce any remarkable haematological, biochemical and histopathological adverse effects in sub-chronic toxicity studies in rats.

## Conclusion

The present study has demonstrated that *D. reticulata* extract exerts antihyperglycemic activity by cytoprotective effect on pancreatic cells, probably via its antioxidant activity, and inhibition of the enzyme α-glucosidase. This study also provides sub-chronic toxicity data to corroborate clinical use of this plant as an alternative antidiabetic agent.
